# Reviewed article: Filho ZVN, Silva NC, Fé RASS, Lourenço MAG, Pazinatto RB, Cabral AEA, Melo LA. Lifestyles associated with complete tooth loss in elderly people in Brazil, 2019. Epidemiol Serv Saude. 2025:34;e20240614

**DOI:** 10.1590/S2237-96222025v34e20240614.b

**Published:** 2025-05-12

**Authors:** Antonio Carlos Faria

**Affiliations:** FOUSP, Odontologia Social

**Table:** 

Rating	Excellent	Good	Average	Below average	Poor
Interest		✓			
Quality		✓			
Originality		✓			
Overall		✓			

**Table:** 

Questionnaire	Yes	No	Not applicable
Does the manuscript contain new and significant information to justify publication?	✓		
Does the Abstract (Summary) clearly and accurately describe the content of the article?	✓		
Is the problem significant and concisely stated?	✓		
Are the methods described comprehensively?	✓		
Are the interpretations and conclusions justified by the results?	✓		
Is adequate reference made to other work in the field?	✓		

## Manuscript Structure:

Length of article is: Adequate

Number of tables is: Adequate

Number of figures is: Adequate

Please state any conflict(s) of interest that you have in relation to the review of this paper (state “none” if this is not applicable): None

## Comments

Título:

Está compreensível e sintético, e reflete o conteúdo a que se propõe o estudo.

Resumo:

Apresenta os objetivos da pesquisa, descreve a metodologia empregada, indica os principais resultados do estudo e escreve as conclusões da pesquisa.

Introdução:

Evidencia o propósito do estudo, justificativas e o(s) objetivo(s) da pesquisa ou questão norteadora, e apresenta sequência lógica das ideias no texto?

Metodologia:

O método empregado é adequado ao tipo de estudo realizado, é escrito de forma clara e completa, descreve o grupo estudado adequadamente, descreve o processo de coleta de dados e os instrumentos utilizados na pesquisa de forma clara e coerente com o tipo de estudo, explicita a análise de dados de forma apropriada ao estudo.

Resultados e Discussão:

Apresenta uma descrição dos resultados de forma clara e objetiva, os resultados correspondem aos objetivos propostos, oferece descrição de resultados necessária para sustentar as conclusões da pesquisa, as tabelas estão em harmonia e construídos de forma adequada, a discussão é pertinente e consistente, confrontam os dados do presente estudo aos de outros trabalhos.

Conclusão:

São claras e concisas, e são coerentes com o desenvolvimento e os resultados do estudo.

Referências:

São pertinentes e atualizadas.

